# Removal of Pulse Artefact from EEG Data Recorded in MR Environment at 3T. Setting of ICA Parameters for Marking Artefactual Components: Application to Resting-State Data

**DOI:** 10.1371/journal.pone.0112147

**Published:** 2014-11-10

**Authors:** Eleonora Maggioni, Jorge Arrubla, Tracy Warbrick, Jürgen Dammers, Anna M. Bianchi, Gianluigi Reni, Michela Tosetti, Irene Neuner, N. Jon Shah

**Affiliations:** 1 Institute of Neuroscience and Medicine 4, INM 4, Forschungszentrum Jülich, Jülich, Germany; 2 Department of Electronics, Information and Bioengineering, DEIB, Politecnico di Milano, Milano, Italy; 3 Bioengineering Lab, Scientific Institute IRCCS E. Medea, Bosisio Parini, Italy; 4 Department of Psychiatry, Psychotherapy and Psychosomatics, RWTH Aachen University, Aachen, Germany; 5 Laboratory of Medical Physics and Magnetic Resonance Technologies, IRCCS Fondazione Stella Maris, Pisa, Italy; 6 JARA – BRAIN – Translational Medicine, RWTH Aachen University, Aachen, Germany; 7 Department of Neurology, Faculty of Medicine, JARA, RWTH Aachen University, Aachen, Germany; University of Maryland, College Park, United States of America

## Abstract

Simultaneous electroencephalography (EEG) and functional magnetic resonance imaging (fMRI) allow for a non-invasive investigation of cerebral functions with high temporal and spatial resolution. The main challenge of such integration is the removal of the pulse artefact (PA) that affects EEG signals recorded in the magnetic resonance (MR) scanner. Often applied techniques for this purpose are Optimal Basis Set (OBS) and Independent Component Analysis (ICA). The combination of OBS and ICA is increasingly used, since it can potentially improve the correction performed by each technique separately. The present study is focused on the OBS-ICA combination and is aimed at providing the optimal ICA parameters for PA correction in resting-state EEG data, where the information of interest is not specified in latency and amplitude as in, for example, evoked potential. A comparison between two intervals for ICA calculation and four methods for marking artefactual components was performed. The performance of the methods was discussed in terms of their capability to 1) remove the artefact and 2) preserve the information of interest. The analysis included 12 subjects and two resting-state datasets for each of them. The results showed that none of the signal lengths for the ICA calculation was highly preferable to the other. Among the methods for the identification of PA-related components, the one based on the wavelets transform of each component emerged as the best compromise between the effectiveness in removing PA and the conservation of the physiological neuronal content.

## Introduction

The combination of electroencephalography (EEG) and functional magnetic resonance imaging (fMRI) can provide a non-invasive comprehensive view of brain activity with high temporal (EEG) and spatial (fMRI) resolution. The EEG technique gives a measure of the synchronized electrical activity of large populations of neurons. Despite its high temporal resolution, which is in the order of tens of milliseconds, the EEG suffers from the spatial inverse problem, related to the difficulty in inferring the spatial location of neuronal sources in the brain from the potentials recorded at scalp level [1], [2].

The fMRI technique based on blood oxygen level-dependent (BOLD) contrast gives information about the hemodynamic processes associated with the neuronal activity. The BOLD measure is extended to the whole brain and has a spatial resolution in the order of mm, but suffers from an ill-posed temporal problem, as it is hard to extract the timings of events that caused the measured hemodynamic modifications [3].

As the strengths and weaknesses of the two approaches are complementary, the simultaneous recording of EEG and fMRI holds great promise for cognitive neuroscience. Among the possible applications, it is worth mentioning the pre-surgical evaluation for epileptic diseases [4], the investigation of neurovascular coupling [5], [6], [7] and connectivity studies [8], [9], [10].

An area of increasing interest is the analysis of EEG and BOLD signals during resting wakefulness. The spontaneous electrophysiological activity exerts a large influence on sensory, cognitive and motor-driven processes [11], [12] and contributes to the total variance of brain electrical activity much more than the evoked/event-related responses [13]. Several fMRI studies showed the presence of multiple specific functional large-scale networks during rest, the so-called Resting State Networks (RSNs). In addition to the default mode network, i.e. a cohesive network supporting a default mode of brain function that appears deactivated during cognitive tasks [14], functional connectivity during rest has been identified for the motor system [15], the language system [16], the attention system [17] and the working memory system [18].

Despite growing knowledge of BOLD RSNs, their underlying electrophysiological signature is still a matter of discussion. One of the main topics to clarify is how the coherent slow fMRI hemodynamic fluctuations are coupled to the fast neuronal activity recorded with EEG. However, a meaningful exploitation of EEG-fMRI information relies on good data quality, especially in the case of resting-state applications. Indeed, while in the study of event-related brain response the interesting information is usually restricted to a group of channels and to specific intervals and is known a priori, in resting-state analysis the global state of the brain is of interest.

Despite the potential advantages of EEG and fMRI integration, its main concern regards the removal of artefacts from the EEG signal recorded in the magnetic resonance (MR) environment. The main artefact affecting the EEG signal is the gradient artefact (GA), caused by the switching of magnetic field gradients required for MR image acquisition. Its amplitude can be up to 100 times larger than the original EEG signal, but since it occurs at fixed time intervals, it is easily removable by subtracting an average GA template from the EEG signal at the channel level [19]. A second type of artefact is indirectly related to cardiac activity and is referred to as pulse artefact (PA). Although the PA amplitude is smaller than that of GA, its removal is more challenging. Indeed, the PA characteristics vary not only across subjects, but also within each subject, as they are non-stationary over space and time. Three factors mainly contribute to PA: first, a ballistic effect is considered to be caused by pulsatile body motion, probably due to the acceleration and abrupt reversal in blood flow in the aortic arch [20]. The movement of electrically conductive material in a static magnetic field leads to electromagnetic induction; therefore, the body’s pulsatile movement causes electromotive forces (EMFs) in the EEG recording system, which in turn affect the registered EEG signal. Additional EMFs are caused by a slight rotation of the head, probably produced by changes of pulsatile blood flow momentum in the cranial arteries [21]. The third main contribution to PA is given by the Hall effect, related to the movement of a conductive fluid (blood) in a static magnetic field which induces electrical potentials recorded at the scalp level [22].

The combination of these factors increases the spatial and temporal complexity of the PA. Up to now, several methods have been proposed for its removal. A first group of techniques operates at the channel level by subtracting from each EEG channel a template of the artefact. There are two common ways to estimate such a template. The averaged artefact subtraction method (AAS) [19] uses as a template a dynamic average of the artefact across its occurrences; more often applied is the optimal basis set method (OBS), which estimates the template using the first (usually 3) principal components of the signal corresponding to PA intervals [23]. Although both of them remove the majority of the artefact, none of them is able to correct the EEG signal completely. As an alternative to channel-based techniques, blind source separation (BSS) techniques have been proposed, among which independent component analysis (ICA) [24] is most commonly used. ICA is used to remove EEG artefacts due to eye blinking or movements [25], in particular those related to the MR environment [26], [27], [10]. ICA decomposes the signal into components that are maximally independent over time; following the assumption that PA sources are independent from neuronal ones, ICA appears to be a suitable technique for retrieving the underlying neuronal information.

However, for the ICA decomposition to be meaningful, the sources should be stationary in space, and often this is not the case with EEG signals. Indeed, not only the spatial topography of PA contribution changes during the cardiac cycle, but the neuronal signals themselves can also be strongly non-stationary over time. Although the ability of ICA to remove PA has been confirmed in more than one work [26], [10], in the literature there are also reported cases of poor ICA performance [28], [29], [30]. The unmet requirement of stationariness could be one of the reasons for the possible failure of ICA algorithm to remove PA. Besides that, the tuning of ICA parameters and the identification of the PA-related independent components (ICs) are challenging.

Recently it was proposed to apply OBS before ICA in order to help in meeting the ICA assumptions and to check if the ICA performance could improve if a reduced amount of artefact was present. The OBS-ICA combines the strengths of both approaches and was confirmed capable of producing satisfactorily improved corrections [30], [31], compared with the single techniques. Nevertheless, the ICA correction entails the risk of deteriorating the EEG signal; the ICA step is performed on a signal already subjected to OBS and less contaminated by artefacts than previously, making the PA contribution in the resulting components less noticeable. This makes the selection of artefactual components a very delicate task. Such selection steps can be performed either by manually inspecting the components (e.g. [32]) or by using semi-automatic or automatic methods. Although several research groups performed the correction by manually selecting the PA-related components [33], [34], the manual approach relies significantly on the user’s experience and without a proper training it cannot be recommended as a routine procedure. Among the automatic or semiautomatic selection criteria, the most common ones look either at the amount of correlation that the ICs share with the electrocardiographic (ECG) signal or a PA template [26] or at the ICs variance [35].

Although Vanderperren et al., [31] inspected the effects of several PA correction methods on the quality of visual event-related potentials (ERPs), up to now the impact of different PA corrections on resting-state data has not been sufficiently investigated. In these data, the information of interest is largely unknown; therefore optimal cancelling of EEG artefacts is extremely important.

Starting from the assumption that OBS-ICA has the potential to improve the quality of EEG signal retrieval [30], [31], the current work is focused on this combined approach and aims at defining an appropriate time interval for ICA calculation and IC selection criteria, as applied to resting-state EEG data recorded at 3T. In particular, two time intervals for ICA calculation were compared, together with four criteria for marking the artefactual components. The different methods were evaluated in terms of their capability to 1) reduce the amount of PA and 2) preserve the information of interest that for the sake of simplicity was identified as the alpha rhythm in the occipital channels or more generally any contribution unlocked to the PA. The comparison was performed on a group of 12 healthy volunteers who underwent EEG-fMRI acquisition during two separate periods of rest interleaved by a cognitive task. The performance of each ICA correction was tested on both the resting-state recordings separately. A comparison between the results of the ICA corrections on the two groups of datasets was performed with the aim of assessing their reliability and reproducibility.

## Materials and Methods

### Subjects

Twelve healthy right-handed volunteers with no history of neurological disorders took part to the study (9 males, mean age = 27.7 yrs, standard deviation = 6.6 yrs). All of them signed a written informed consent to the protocol, in accordance with local ethical committee guidelines.

### EEG-fMRI data acquisition

All EEG data were recorded simultaneously with fMRI recordings in a Siemens 3T Trio MR scanner (Germany). EEG data were acquired using an MR-compatible EEG system (Brain Products, Gilching, Germany). The EEG cap (BrainCap MR, EasyCap GmbH, Breitbrunn, Germany) included 63 scalp electrodes distributed according to the 10–20 system and one additional ECG electrode placed on the participants’ back. EEG signals were acquired relative to an FCz reference, with the ground in correspondence of Iz (10-5 electrode system). The EEG data were sampled at 5000 Hz, with a band-pass filtering of 0.016–250 Hz. The impedance at each electrode was kept lower than 10 kΩ.

### Protocol

The study protocol was approved by the local human subjects review board at RWTH Aachen University and was carried out in accordance with the Declaration of Helsinki. Two phases of rest lasting 6 minutes (i.e. 180 fMRI scans) were separated by 3 runs of a visual oddball task lasting 10 minutes and 8 seconds (i.e. 304 fMRI scans) per run. During resting wakefulness the subjects were asked to keep their eyes closed. The analysis was performed only on the EEG resting-state recordings, the data from the visual oddball task are presented elsewhere [36], [37].

### EEG data processing

**Figure 1:**
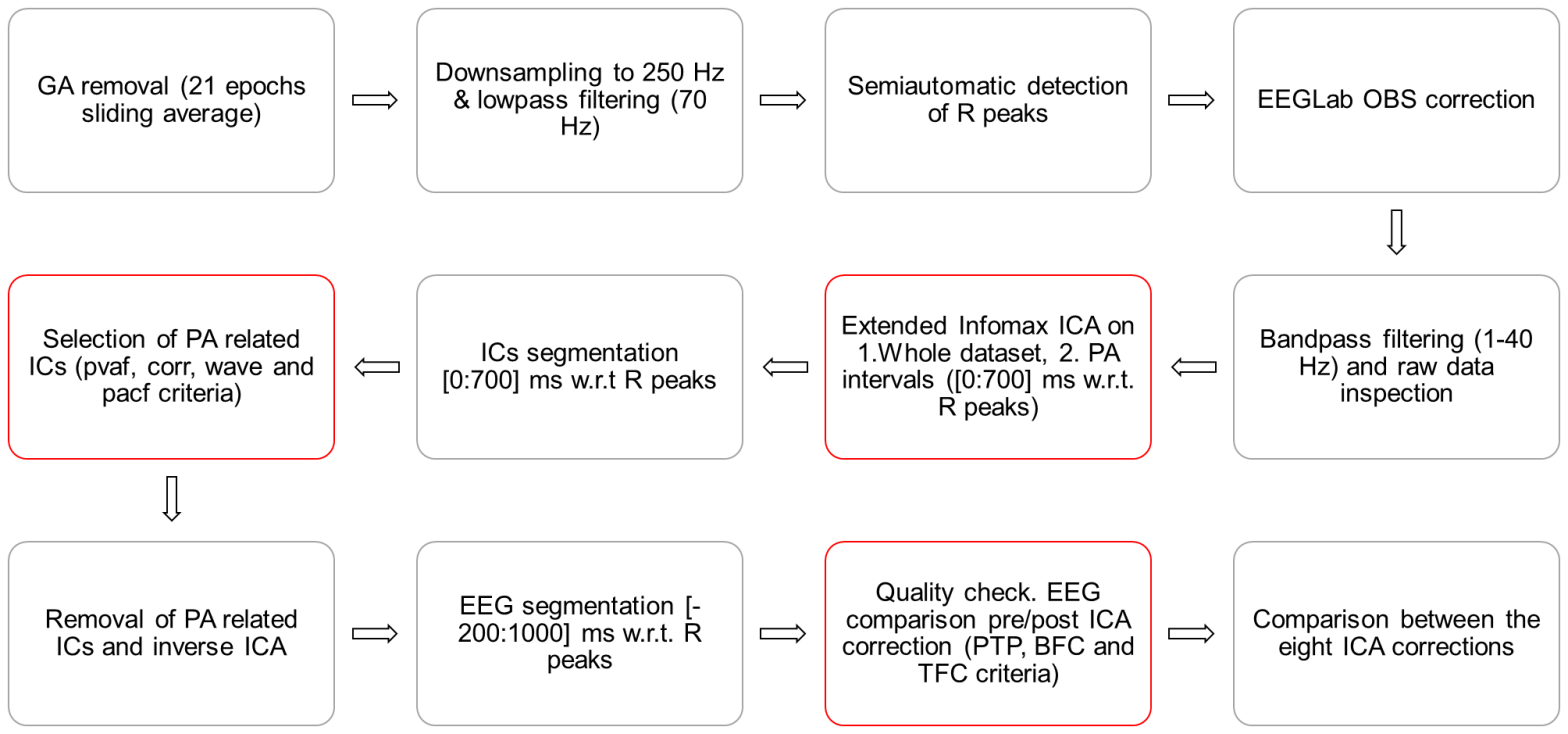
Schematic illustration of the EEG data processing.

A schematic illustration of the entire processing stream is provided in Figure 1. The EEG data were first cleaned by GA and downsampled to 250 Hz with BrainVision Analyzer 2.0 software (BrainProducts, Gilching, Germany). The imaging artefact was corrected by subtracting from each channel a template created using a sliding average of 21 GA blocks. The R peaks were identified using the specific tool provided by Analizer 2.0 in semi-automatic modality. The first R peak was semi-automatically selected from a well-defined QRS complex. This was used as a template for the identification of all other R peaks, which was performed by the software. The correct position of the R peaks was verified by the user and corrected where necessary. After the R peaks identification, the EEG raw data were exported into Matlab 7.11.0 (R2010b) and the FMRIB plug-in of the EEGLAB toolbox (version 11.0.5.4b) [38] was used to perform the OBS correction, where the default parameters were used, i.e. a basis set of the first 3 principal components was the PA template. The EEG signals were then reimported into Analyzer 2.0, where the extended infomax ICA [39] was applied in order to reject the residual cardiac related artefact. ICA was applied after segmentation of the EEG signal from the fifth fMRI scan onwards. To compute the ICA mixing matrix we used either the whole data (ICA_whole) or epochs lasting from 0 to 700 ms with respect to the R peaks (ICA_R). The components resulting from each ICA calculation were segmented into PA intervals (from 0 to 700 ms w.r.t. the R peaks) and further analyzed. The components to be removed were identified following four different methods; the first three were implemented in Matlab scripts and the fourth was a function of Analyzer 2.0. We evaluated the different ICA parameter settings separately on the two groups of datasets, relative to the resting-state periods preceding (Dataset1) and following (Dataset 2) the cognitive paradigm. The comparison was performed on eight ICA-based methods, resulting from the combination of the two types of ICA calculation (ICA_whole and ICA_R) and the four criteria for selecting the PA-related ICs.

### Selection of PA-related components

#### Variance contribution (pvaf)

**Figure 2:**
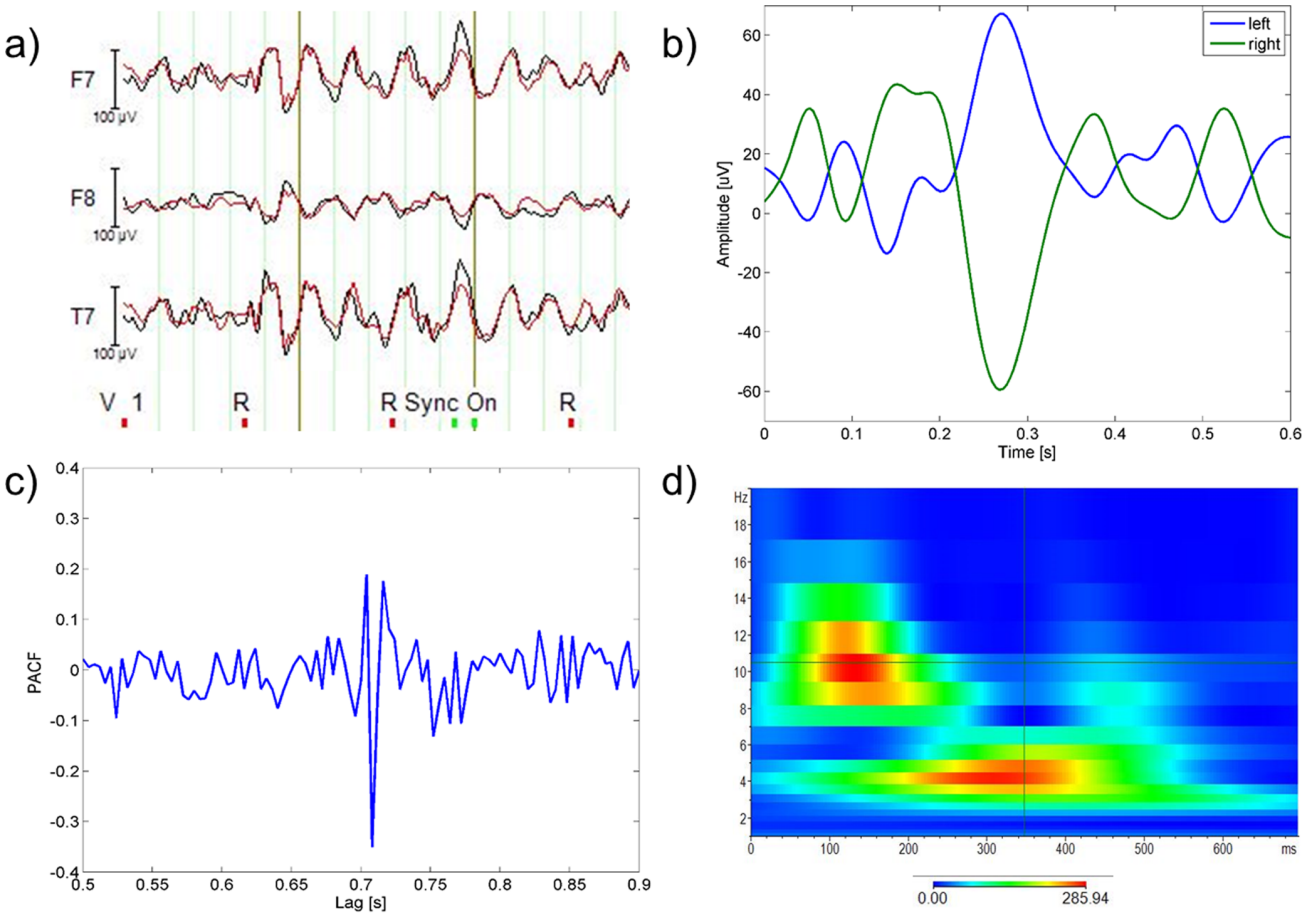
Methods for selection of PA-related components. a) pvaf method: Variance contribution of one exemplar component (IC backprojection in red, original EEG in black), b) corr method: PA templates of one subject (left hemisphere in blue, right one in green), c) pacf method: PACF of one representative IC, d) wave method: wavelets transform (instantaneous amplitude, gabor normalization) of one representative PA template.

Each component was back-projected to the EEG signal space, and the variance of the resulting signal across the PA interval (0–700 ms after R) was calculated and compared to the initial EEG variance during the same interval, following the same procedure described in [31], [35]. The comparison relative to one representative IC is displayed in Figure 2 a). The ICs that explained more than the 2.5% of the initial variance were marked as PA-related and removed.

#### Correlation (corr)

We evaluated the cross-correlation between each IC and two PA templates. Since the cardiac-related artefact was found to change polarity from one side of the head to the other [35], [40], we estimated one template for each hemisphere. Each template was created by averaging the EEG uncorrected signals (before OBS) over the PA intervals and over the left/right EEG channels (the mesial channels were included in both templates). In Figure 2 b) one subject’s templates are plotted as an example. Instead of using an absolute correlation threshold, we used as reference the maximum correlation between each template and the ICs, marking as cardiac-related the ICs whose correlation with one of the templates was higher than the 40% w.r.t. the maximum. The choice of a relative threshold with respect to an absolute one was justified by the differences in correlation coefficients across subjects.

### Partial AutoCorrelation Function (PACF)

Blocks formed by four PA intervals were averaged and the PACF was calculated, similarly to that performed in [31]. The ICs with a peak at R–R distance lag were selected (an exemplar PACF with R–R peak is in Figure 2 c)) and the maximum peak amplitude across these ICs was used as a reference. The ICs with a peak amplitude higher than one third of the maximum were removed.

The thresholds of the automatic selection criteria were chosen on an empirical basis, expressly equal across subjects.

#### Wavelets analysis (wave)

For each IC, a continuous wavelets transform (CWT) was performed in each PA interval, and the CWTs across intervals were averaged. We used the Morlet complex family of wavelets (central frequency = 14.591 Hz, bandwidth = 5.836 Hz) and investigated frequencies going from 1 Hz to 20 Hz with twenty steps in between. Basing on the time-varying frequency content of the PA templates, the ICs having a peak time locked to the R peak between the delta and alpha band were selected and removed. Figure 2 d) shows the CWT of an exemplar PA template. This selection method, which has not been used in previous studies, was created in the attempt to emphasize the frequency contributions time-locked with the cardiac cycle, which characterize the PA-related components. The selection was performed by one person, who was trained on the inspection of components and their time-frequency transforms for two and a half months.

#### Validation criteria

To check the quality of PA removal, EEG epochs from −200 ms to 1 s w.r.t. R peaks, before and after ICA correction, were extracted and compared. We then assessed the performance of the different ICA corrections by means of three different criteria. When the effects of PA correction on the alpha content were examined, only the occipital channels were considered, otherwise all the EEG channels were used to assess the quality of PA correction. In each validation, the quality measures of the eight ICA-based methods were compared through a non parametric Kruskal Wallis (KW) test, for each dataset separately; if significant differences emerged at the group level, the KW statistics were used in a multiple comparison test to extract the pairwise differences. This was followed by two further comparisons, between 1) the four selection methods (across datasets and ICA intervals) and 2) the two ICA intervals (across datasets and selection methods).

#### Peak-to-peak (PTP) value

Assuming that the maximum signal amplitude corresponds to PA occurrence, the ratio between the maximum signal variation after and before ICA correction is proportional to the amount of artefact removed by ICA. This ratio, averaged over all the EEG channels, was therefore used to estimate the effectiveness of the PA correction.

### Batch frequency content (BFC)

Subjects having an evident alpha peak in the mean PSD before ICA were selected (Subj4, Subj9, Subj11 and Subj12) with the aim of checking if the alpha rhythm could be retrieved after ICA correction. For each epoch, we computed the power spectral density (PSD) of the occipital channels with an autoregressive (AR) spectrum. The PA shows a main contribution in the low frequency range (between around 4 and 8 Hz) and an additional one in the alpha range (from 8 to 13 Hz). Since during rest the signal of interest in the occipital channels is mainly in the alpha band, assuming that the neuronal alpha rhythm contributes to the most of the alpha power, a good PA correction should remove as much of the low frequency contribution as possible while maintaining most of the alpha power. For this purpose, we estimated the mean PSD across epochs and occipital channels (O1, O2 and Oz) and looked at the ratio between delta (delta ratio), theta (theta ratio) and alpha (alpha ratio) power after and before ICA. Additionally, we defined a quality coefficient (QC) as the ratio between the alpha ratio and the mean value between delta and theta ratios: such a measure is proportional to the amount of 1) low frequency power cancelled and 2) alpha power preserved.

In addition to the statistical analysis of the QC values (QC_test), a comparison including all subjects and channels was performed (group_test). In this case, the previous assumptions on the alpha contribution were no longer reliable, furthermore no information of interest was expected in the higher frequency bands. Therefore, the quality of correction was only evaluated in terms of the proportion of delta and theta power that was removed.

### Time-varying frequency content (TFC)

We added this validation criterion to provide further details about the ICA correction effects on the signal spectral content. Indeed, the change of frequency power can give ambiguous information, especially if the alpha band is considered. Since the percentages with which PA and neuronal signals contribute to the total alpha power are not known a priori, it is difficult to state whether the physiological alpha rhythm is preserved or not just by looking at the alpha power change. Nevertheless, the alpha temporal properties can help in distinguishing PA alpha from neuronal alpha, since the latter is not temporarily locked to PA occurrence. As a consequence, the modifications in time-varying alpha content induced by ICA can provide further information on the correction quality.

**Figure 3:**
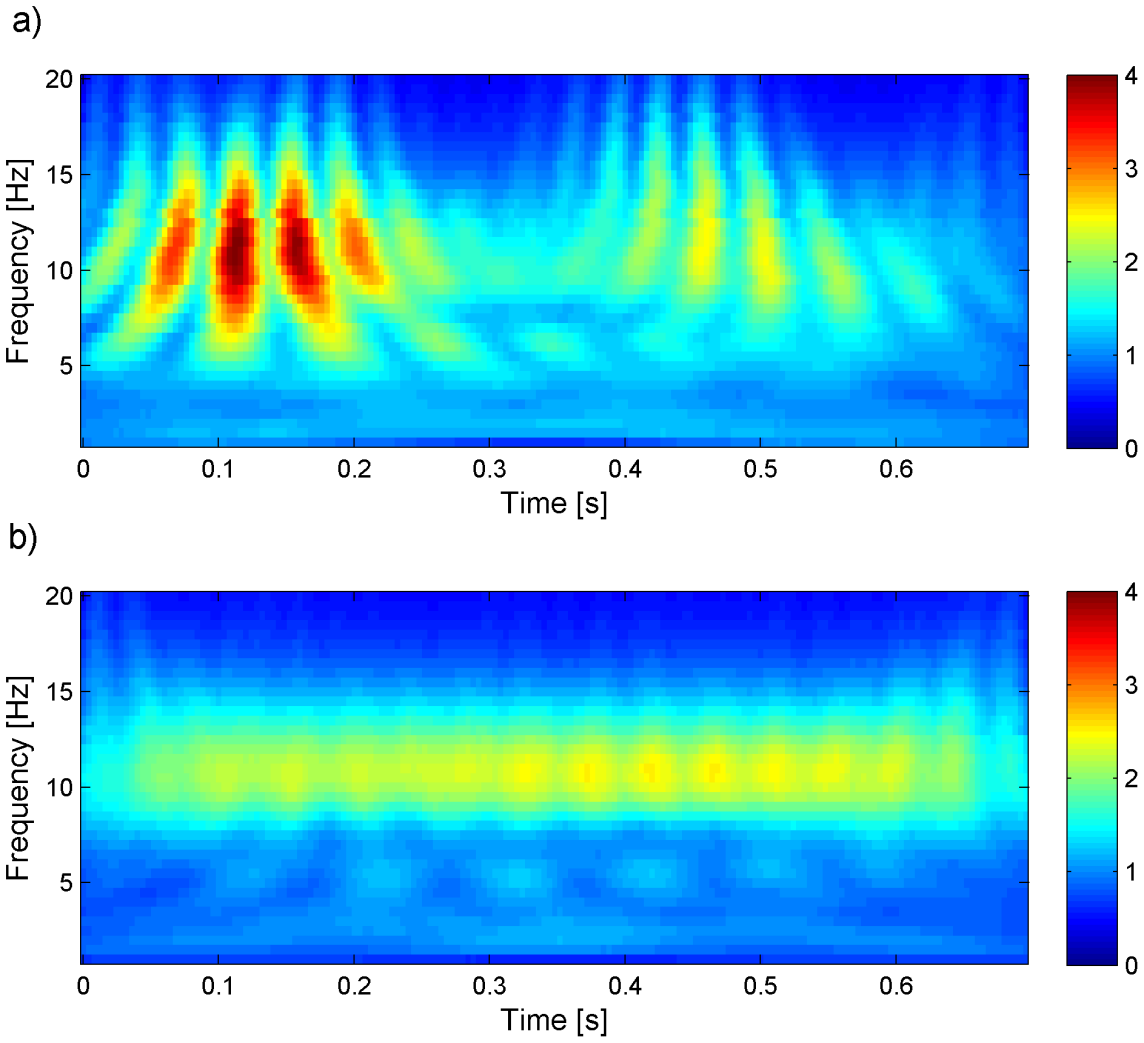
Mean CWT (absolute values) across PA intervals of two independent components of the EEG signal of one representative subject (before ICA correction). One component is artefactual (panel a) and one is physiological (panel b).

More generally, in the entire frequency spectrum a good correction can be assessed by looking at the continuous frequency component (physiological) compared to the PA-locked ones (artefactual), without having any a priori knowledge on 1) their contribution to the total power and 2) the frequency band of interest. The reader can have a better idea of the difference between physiological and artefactual frequency contributions by looking at Figure 3, where the time-frequency transforms of one PA-related and one physiological source of the EEG signal are compared. For each subject and EEG channel we computed the mean CWT across epochs using a Morlet wavelet (central frequency = 0.8125 Hz), before and after ICA correction. Absolute CWT values were considered. We then performed averaging across all subjects and 1) all channels or 2) only the occipital ones: in the latter, we expected to find a continuous alpha contribution, in particular after ICA correction. We emphasized the time-frequency components that were removed from each ICA correction by subtracting the CWT of the corrected signal (CWT_post_) from the CWT of the uncorrected signal (CWT_pre_). After visual inspection of such difference, dubbed CWT_off_, we used its time derivative (averaged over both time and frequency) as metric for the correction quality. Under the hypothesis that low and high values of derivative can be associated to physiological and PA-related frequency components respectively, the selection methods corresponding to higher mean derivatives of CWT_off_ were evaluated better than others.

## Results

Across datasets and ICA intervals, it emerged that the variance-based selection criterion removed less components compared to the others, with 9.2±2.6 (mean ± standard deviation) out of 63 ICs removed, against 19.6±7.3 of the correlation method, 21.4±4.9 of the wavelets method and 20±5.3 of the partial autocorrelation function method. The results of each validation method relative to both the datasets are shown below.

### PTP value

**Figure 4:**
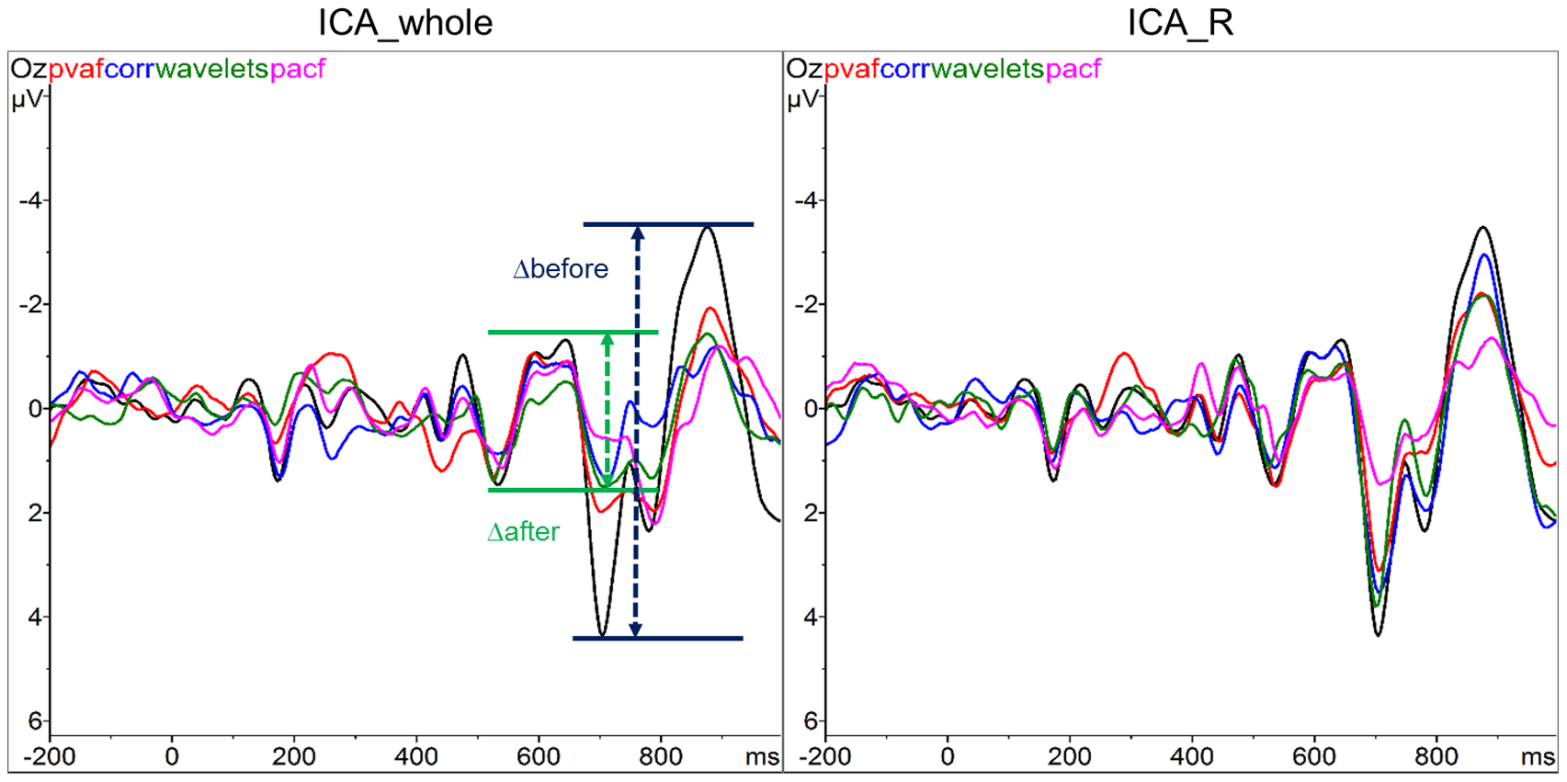
Example of PTP value comparison relative to the Oz channel of one subject. The amplitude ranges of EEG signal before (black curve) and after correction with ICA were compared, using the two ICA calculations (ICA_whole on the left, ICA_R on the right) and the four methods for selection of components (the colour legend is at the top left of each plot).

The PTP value comparison (of which Figure 4 is an example) revealed differences in the eight ICA correction methods in terms of their effectiveness in reducing the PA amplitude. The PTP ratio mean and standard deviation of the eight ICA-based methods on Dataset1 and Dataset2 are listed in Table 1 and Table 2 respectively.

**Table 1:** Peak to peak ratio (mean ± standard deviation across subjects) of each method for ICA correction in Dataset1.

	Calculation on whole data (ICA_whole)				Calculation on PA intervals (ICA_R)			
Correction method	pvaf	corr	wave	pacf	pvaf	corr	wave	pacf
**PTP ratio**	0.7162±0.0984	0.7943±0.1392	0.7904±0.1609	0.7988±0.1609	0.7394±0.0804	0.7968±0.1571	0.7267±0.1982	0.7345±0.1632

**Table 2:** Peak to peak ratio (mean ± standard deviation across subjects) of each method for ICA correction in Dataset2.

	Calculation on whole data (ICA_whole)				Calculation on PA intervals (ICA_R)			
Correction method	pvaf	corr	wave	pacf	pvaf	corr	wave	pacf
**PTP ratio**	0.7069±0.1138	0.8577±0.0698	0.8030±0.1205	0.8006±0.1891	0.7335±0.0798	0.8430±0.1576	0.7842±0.1326	0.8185±0.1712

The findings of the two datasets were in agreement. The ICA calculation on the whole signal combined with the variance contribution method for the selection of PA-related ICs (ICA_whole_pvaf) led to the best results in terms of PTP ratio, because in both datasets the mean percentage of residual PTP associated with this method was minor compared to the others. The KW test between all different methods showed significant differences only in Dataset2 (p<0.02), where the multiple pairwise comparison showed that when the ICA matrix was calculated from the whole dataset, the variance selection method (ICA_whole_pvaf) performed significantly better than the correlation method (ICA_whole_corr) (p<0.05). By comparing the four selection methods across ICA intervals and datasets, significant differences emerged (p<0.01). In particular, the pvaf method performed better than the corr method (p<0.01) and pacf method (p<0.05). Despite the lower PTP ratio, no significant differences were detected with respect to the wave method. The performance of the corr method was poor in comparison to the others. The statistical test between the two ICA intervals showed no significant differences; indeed, the performance of ICA_whole with respect to ICA_R was variable and dependent on the selection method and the dataset under examination.

### BFC

The comparison between the eight ICA-based methods based on their frequency content led to partially conflicting results. Indeed, while the group_test results were in line with the PTP value results, the QC_test provided discordant information with respect to them.

The group_test (including all the subjects and all the EEG channels) confirmed the capability of the pvaf method to remove the low frequency artefactual contribution. The values of the ratio between the delta and theta power after and before the eight ICA corrections are listed in the upper panel of Table 3 (Dataset1) and Table 4 (Dataset2). Significant differences were found between the methods (p<0.01 in both datasets). In Dataset1, the pvaf_whole method removed significantly more low frequency (LF) power than corr_whole (p<0.01), wave_whole (p<0.03), pacf_whole (p<0.01) and corr_R (p<0.04) methods. In Dataset2, the pvaf_whole method removed significantly more LF power than corr_whole (p<0.01), wave_whole (p<0.01), corr_R (p<0.03) and pacf_R (p<0.04) methods. Summarizing across datasets and intervals used for ICA calculation, the selection based on variance led to the greatest removal, followed by the wave, pacf and corr selection methods. The KW analysis showed a significant difference among these methods (p<0.01), with the pvaf method significantly different from the other three (p<0.01). No significant differences were identified between the two intervals for ICA calculation (ICA_whole and ICA_R).

**Table 3:** Spectral coefficients (mean ± standard deviation across subjects) of each ICA correction in Dataset1.

	Calculation on whole data (ICA_whole)				Calculation on PA intervals (ICA_R)			
Correction method	pvaf	corr	wave	pacf	pvaf	corr	wave	pacf
***Group_test***								
**Delta+Theta ratio**	0.8372±0.1368	1.3522±0.4610	1.2962±0.3645	1.3357±0.3695	0.9079±0.1442	1.2698±0.4199	1.0936±0.2889	1.1386±0.3126
***QC_test***								
**QC**	0.8238±0.4163	1.1722±0.5797	1.1218±0.5721	0.9092±0.3410	0.6952±0.2526	0.9668±0.6250	1.0670±0.5533	0.8598±0.4232
**Alpha ratio**	0.4113±0.2659	0.7878±0.1701	0.6297±0.2516	0.6793±0.1705	0.3655±0.1782	0.5219±0.3162	0.5723±0.2311	0.5530±0.2979
**Delta ratio**	0.4872±0.1151	0.7576±0.2400	0.6193±0.1429	0.8290±0.2609	0.5675±0.0727	0.6118±0.1208	0.6363±0.2499	0.6831±0.2063
**Theta ratio**	0.4383±0.2124	0.7241±0.2298	0.5421±0.2190	0.7655±0.2594	0.4592±0.1418	0.4784±0.2280	0.4952±0.1304	0.5903±0.2122

*Group_test:* delta+theta ratio (delta+theta power after ICA correction divided by delta+theta power before ICA correction), averaged over all subjects and channels. *QC_test:* QC, alpha ratio, delta ratio and theta ratio averaged over the occipital channels of the four subjects with alpha rhythm.

**Table 4:** Spectral coefficients (mean ± standard deviation across subjects) of each ICA correction in Dataset2.

	Calculation on whole data (ICA_whole)				Calculation on PA intervals (ICA_R)			
Correction method	pvaf	corr	wave	pacf	pvaf	corr	wave	pacf
***Group_test***								
**Delta+Theta ratio**	0.8859±0.1667	1.5801±0.2256	1.4314±0.3079	1.3229±0.3672	0.9595±0.1271	1.3672±0.3622	1.3589±0.2012	1.3660±0.4060
***QC_test***								
**QC**	0.6957±0.2535	0.8203±0.1961	0.8536±0.2019	0.7822±0.4141	0.8010±0.2840	0.8481±0.1498	0.9987±0.2165	1.0207±0.4961
**Alpha ratio**	0.3628±0.1291	0.7445±0.1373	0.5980±0.2921	0.4855±0.2369	0.4604±0.1916	0.7828±0.1094	0.5463±0.2293	0.7112±0.2951
**Delta ratio**	0.6399±0.2385	0.9810±0.1830	0.7149±0.2566	0.6875±0.2113	0.5963±0.2051	0.9843±0.1789	0.5974±0.2190	0.7371±0.2399
**Theta ratio**	0.4496±0.1034	0.8640±0.1053	0.6329±0.2717	0.6370±0.2676	0.5694±0.2446	0.8772±0.0971	0.5135±0.1741	0.7176±0.2886

*Group_test:* delta+theta ratio (delta+theta power after ICA correction divided by delta+theta power before ICA correction), averaged over all subjects and channels. *QC_test:* QC, alpha ratio, delta ratio and theta ratio averaged over the occipital channels of the four subjects with alpha rhythm.

The results of the QC_test (considering only the occipital channels of the four subjects with alpha peak) are listed (mean ± standard deviation) in Table 3 and Table 4 for Dataset1 and Dataset2 respectively. These tables include the ratio between the delta, theta and alpha power after and before ICA correction. The KW analysis performed on the eight methods with each of the computed measures (QC, delta_ratio, theta_ratio and alpha_ratio) showed no significant differences.

**Figure 5:**
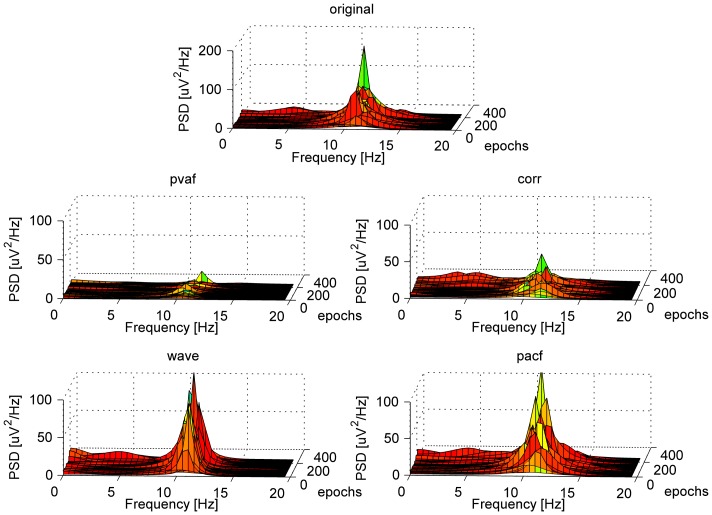
AR power spectral density across EEG epochs relative to the occipital channels of one representative subject, before and after ICA correction with the four selection methods. The spectral contents were averaged across the PA intervals (ICA_whole and ICA_R).

Nevertheless, we could identify differences in the eight ICA-based methods’ performance. In contrast with the PTP validation, the selection method based on variance contribution was associated with the lowest QC, regardless of the interval used for ICA calculation: the pvaf method reduced the LF power more than the others, but it also cancelled the majority of the alpha power. When looking at both datasets, the other selection methods led to good and comparable results in terms of QC. In some cases, the corr and pacf methods preserved a higher percentage of alpha power than the wave method, but in such cases they were less effective in removing the LF contribution. By comparing the two ICA intervals (across datasets and selection methods) and the four selection methods (across datasets and ICA intervals) separately, no significant differences emerged. However, the wave selection method had the highest mean QC value (QC = 1.01±0.4), immediately followed by corr (QC = 0.95±0.42) and then by pacf (QC = 0.89±0.39) and pvaf (QC = 0.75±0.28) ones respectively. The wave method removed more alpha power compared to the pacf and corr ones, but the method also removed more components in the delta and theta frequency ranges (data not shown). Figure 5 shows for one representative subject the spectral content across PA epochs, before and after ICA corrections with the four selection methods.

### TFC

The visual inspection of the CWT_off_ of each ICA-based method, representing the time-varying frequency components removed at the group level, allowed us to easily discriminate between poor and good corrections. The qualitative and quantitative comparisons based on CWT_off_occ_ (occipital channels) and CWT_off_all_ (all channels) led to results that were in line with the findings of the previous QC_test: indeed, they confirmed the higher reliability of the wave method with respect to the pvaf one in preserving the information of interest.

The results of the quantative comparison based on the mean derivative (MD) of the CWT_off_ are described hereinafter. The MD values are listed in Table 5 (Dataset1) and Table 6 (Dataset2). In the analysis of CWT_off_all_, the KW statistics showed significant differences between the eight ICA corrections (p<0.01 for both datasets). Looking at the pairwise comparisons, in Dataset1, ICA_R_wave method performed significantly better than ICA_R_corr method (p<0.03), whereas no significant pairwise differences emerged in Dataset2. No significant differences emerged from the comparison between the two ICA intervals (across datasets and selection methods), whereas the comparison between the four selection methods showed significant differences (p<0.01), with the corr and pacf methods significantly worse than the wave and pvaf methods (p<0.01). In particular, the wave method was first-ranked, followed by pvaf, pacf and corr methods.

**Table 5:** Mean derivative of CWT_off_ (time-frequency transform of the EEG signal removed by PA correction) corresponding to each ICA-based method in Dataset1 (mean ± standard deviation across subjects).

	Calculation on whole data (ICA_whole)				Calculation on PA intervals (ICA_R)			
Correction method	pvaf	corr	wave	pacf	pvaf	corr	wave	pacf
**MD of CWT_off_all_**	0.7504±0.1979	0.4559±0.0837	0.8029±0.2495	0.3909±0.1424	0.7424±0.1423	0.3482±0.1233	0.9246±0.2425	0.4986±0.1497
**MD of CWT_off_occ_**	0.8077±0.3595	0.4004±0.1047	0.8685±0.4561	0.4712±0.1410	0.8595±0.2805	0.4803±0.2091	0.9578±0.4067	0.6499±0.1478

CWT_off_all_: averaged over all channels. CWT_off_occ_: averaged over occipital channels.

**Table 6:** Mean derivative of CWT_off_ (time-frequency transform of the EEG signal removed by PA correction) corresponding to each ICA-based method in Dataset2 (mean ± standard deviation across subjects).

	Calculation on whole data (ICA_whole)				Calculation on PA intervals (ICA_R)			
Correction method	pvaf	corr	wave	pacf	pvaf	corr	wave	pacf
**MD of CWT_off_all_**	0.7537±0.1742	0.2467±0.0702	0.8189±0.3398	0.2689±0.0716	0.6963±0.0909	0.3607±0.1526	0.8051±0.3505	0.3937±0.0799
**MD of CWT_off_occ_**	0.7338±0.4569	0.2868±0.1347	0.9143±0.4511	0.4260±0.1825	0.6402±0.2437	0.3316±0.1878	0.8638±0.5203	0.4326±0.2331

CWT_off_all_: averaged over all channels. CWT_off_occ_: averaged over occipital channels.

Similar results emerged from the analysis of CWT_off_occ_, where significant differences were found between the eight ICA corrections in Dataset1 (p<0.03), but not within the single pairs of methods. Again, significant differences emerged between the four selection methods but not between the two ICA intervals (p<0.01). The rank was the same as in CWT_off_all_. The pairwise comparison showed that the corr method was significantly worse than pvaf and wave methods (p<0.01), while the pacf method was just worse than the wave method (p<0.02).

These quantitative findings were confirmed by the visual inspection of CWT_off_ and CWT_post_ (especially the ones relative to occipital channels), from which emerged the capability of the wave method to remove the time-varying alpha, locked to the PA, while leaving intact the continuous alpha. The visual inspection proved the poor performance of the corr method, which left the time-varying contribution related to the PA untouched, and confirmed the tendency of the pvaf method to remove information of interest. The pacf method performed better than corr but worse than wave and pvaf methods.

**Figure 6:**
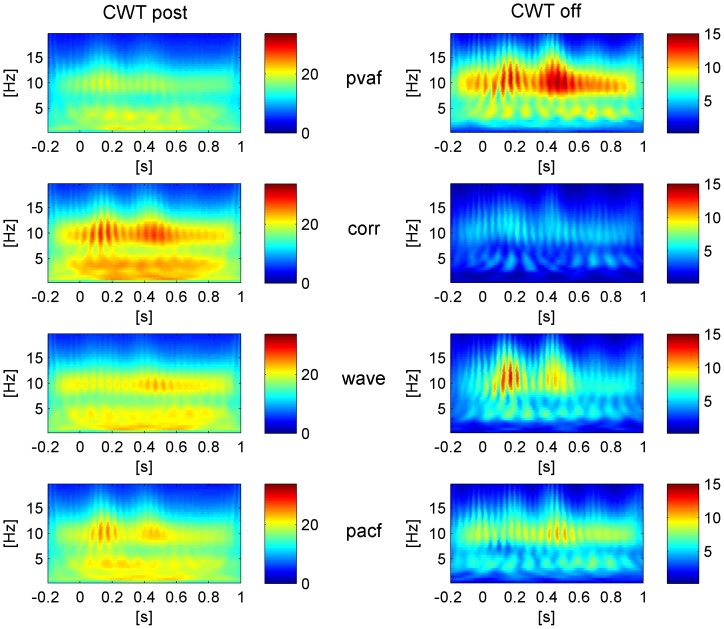
Group level based CWT (absolute values). Left: CWT of the EEG signals after correction (CWT_post_) with the four selection criteria, averaged across R epochs and occipital channels. Right: CWT of the EEG signal removed by each ICA correction (CWT_off_), averaged across R epochs and occipital channels. The shown correction is relative to ICA calculation based on whole data (ICA_whole).

**Figure 7:**
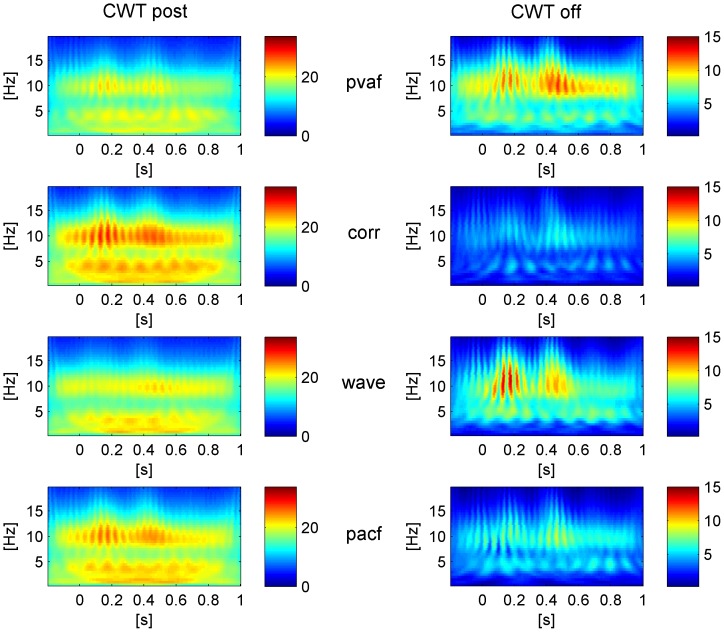
Group level based CWT (absolute values). **Left: CWT of the EEG signals after correction (CWT_post_) with the four selection criteria, averaged across R epochs and occipital channels**. Right: CWT of the EEG signals removed by each ICA correction (CWT_off_), averaged across R epochs and occipital channels. The shown correction is relative to ICA calculation based on the PA intervals (ICA_R).

The CWT_post_occ_ and the CWT_off_occ_ of the eight ICA corrections, relative to Dataset1, are shown as example in Figure 6 (ICA_whole) and Figure 7 (ICA_R). Whichever ICA interval was used, the CWTs after ICA correction (on the left panels) show how the wavelet method left the most continuous alpha contribution, although it removed the low frequency artefactual contribution less than the pvaf method. Further confirmation can be found by looking at the CWT_off_occ_ (right panels), displaying that 1) the wave method removed only the PA-related alpha and 2) the pvaf method removed the PA more than the others but together with a portion of continuous alpha power.

## Discussion

The main objective of the present study was to identify the optimal ICA parameters for the removal of PA from EEG data recorded in an MR environment, after OBS correction. Since our interest was the analysis of spontaneous brain activity with EEG and fMRI, we discussed the effects of different ICA parameter settings on resting-state EEG data recorded at 3T. We compared two intervals for the calculation of the ICA mixing matrix, 1) the entire signal and 2) the PA intervals, together with four methods for selecting the PA-related ICs, based on their 1) contribution to the artefact variance, 2) correlation with PA templates, 3) wavelets transform and 4) partial autocorrelation function. The quality of the EEG cleaning was assessed by looking at the changes occurring after ICA correction in the EEG signal around the R peaks (from −200 ms to 1 s after it). Three different criteria were considered, based on the EEG 1) peak to peak amplitude, 2) batch spectral content and 3) time-varying spectral content. The comparison was performed on two groups of datasets relative to the same 12 subjects: the general agreement between the outcomes of the two comparisons highlighted the reliability of each ICA correction, whose performances were usually reproducible across datasets. The selection of PA-related ICs based on their wavelets transform emerged as the best compromise between the amount of removed PA and the preservation of the neuronal alpha content.

### Comparison with previous studies

To the best of our knowledge, this is the first study comparing different ICA corrections in terms of their capability to retrieve resting-state data information from data measured in an MR environment. Indeed, the widespread comparison between OBS, ICA and OBS-ICA methods described in [31] investigated the PA removal quality in ERP data from visual tasks, following and extending the comparative analysis on auditory ERPs performed in [30]. Grouiller et al. [28] evaluated algorithms for the removal of imaging and cardiac artefacts looking at the goodness of retrieval of the alpha rhythm modulation from a block paradigm and the correct identification of interictal spikes; despite the similar application, they compared ICA to other methods without investigating the details of parameter setting.

In our work, among the several methods proposed to remove the PA, we focused on OBS-ICA combination, found to be capable of improving the correction performed separately by each of the two techniques [30], [31]. Despite this potentiality, the additional use of ICA after OBS involves the risk of affecting the quality of the underlying neuronal signal. In resting-state data, such risk is especially high: since the information of interest is global and not always predictable, the discrimination between neuronal and PA-related ICs is especially challenging. Our main aim was therefore to identify the most appropriate method for selecting the PA-related components. We added the new selection method based on a visual inspection of the ICs wavelets transform averaged across the R epochs to the commonly used criteria based on correlation with a PA template, variance contribution to the PA and autocorrelation of the ICs. Our new selection method represents the main finding of this paper, since it emerged as a valuable criterion for marking the PA-related components.

### Validation criteria and main findings

The quality of PA correction was evaluated from different perspectives. The PTP comparison looked at the change in the maximal variation of the EEG signal due to ICA correction, assuming the PTP value to correspond with PA occurrence. However, this criterion provided information regarding the amount of PA removal only, whereas the validations based on the frequency content change were also potentially sensitive to the deterioration of the signal of interest; in this application, the latter was identified as the alpha rhythm clearly visible in the occipital channels of four subjects (BFC validation, QC_test), and more in general as the frequency components unlocked to PA occurrence (TFC validation).

The BFC criterion inspected the modifications induced by ICA correction to the EEG batch frequency spectrum, giving a quantitative measure of the power change in each band of interest (delta, theta, alpha). This approach assumes that the PA spectrum is characterized by peaks at heart-rate frequency and its harmonics [40]. We also assumed that the main PA contribution would occur in the low frequency range and a smaller one in the alpha range, according to the frequency content of the PA templates of all subjects.

A first quality check was performed on the occipital channels of the four subjects with a visible alpha peak, where the PA contribution to the alpha band was estimated to be less than the neuronal one. We therefore considered the quality of each ICA correction as proportional to the percent of 1) removed low frequency power (delta and theta bands) and 2) preserved alpha power. For this purpose, we defined a quality coefficient as the ratio between the former and the latter. With respect to the PTP comparison, this validation accounted for both the artefact removal and the recovery of the underlying information; on the other hand, we could not define a range of QC values determining a good correction, since the proportion of alpha power related to PA was unknown. In this application, we considered the ICA corrections associated with higher QC as better than the others, but in doing this we discarded the presence of a PA contribution to the alpha power.

To make the statistical analysis stronger, we additionally evaluated the change in batch frequency power in all subjects and channels. However, the assumptions on the predominance of alpha physiological content in the total alpha were not valid anymore, therefore we only investigated the amount of low frequency power that was removed.

In summary, the BFC validation provided more detailed information with respect to the PTP comparison, but was limited by the impossibility to distinguish between PA-related and physiological contributions in the same frequency band. The investigation of the time-frequency information proved to be more suitable for this purpose.

The TFC criterion inspected the modifications induced by ICA-based methods to the EEG time-varying frequency content using the CWTs. Since the PA-related frequency content was locked in time to PA occurrence, while the physiological one was independent, we evaluated the quality of cleaning in terms of the capability to preserve the long lasting contribution and cancel the one locked in time to the PA occurrence. In particular, we analyzed the time-frequency components that were removed by each ICA correction, both visually and quantitatively. This criterion helped in discriminating the neuronal and PA-related frequency contribution and provided further valuable information for the quality assessment.

It is worth mentioning that the two frequency-validation criteria (BFC and TFC) are specific to our work with respect to the previous methodological comparisons and proved to be extremely useful in the assessment of the effects of each ICA correction on the signal of interest. The results of the comparative analysis strengthen the need to consider different factors when assessing the quality of PA correction. While the validation criteria agreed on the similar performance of the two intervals for ICA calculation, their findings on the four selection methods were more discordant. Indeed, the PTP criterion led to conclusions that were partially in disagreement with the other validation criteria. The former showed that the selection of components based on variance led to the greatest PA removal and marked it as the best selection method. On the contrary, the QC and TFC validations revealed that this criterion removed a higher percent of alpha power than the other selection methods, including part of the neuronal alpha signal. The visual inspection and quantitative TFC comparison revealed the inability of the variance selection method to discriminate between neuronal and PA-related components and the higher reliability of the wavelets selection method to this end.

Summarizing the results of the three validation criteria across the two datasets, the selection of the PA-related ICs based on their wavelets transform emerged as the best compromise between the reduction in the PA amplitude and the preservation of the underlying resting-state information. Indeed, in most cases looking at the time varying frequency content allowed us to distinguish easily between neuronal and artefactual components, given the differences in their temporal properties. The wavelets-based selection criterion represents a novelty of our comparison with respect to previous ones. Among the other selection methods, the one based on the ICs variance showed a more robust performance compared to the correlation and autocorrelation ones. The pvaf method may represent a good choice in cases where an automatic method is required, such as when a big dataset has to be analyzed in a short time, or in datasets recorded in conditions different from rest. In future applications, it could be worth combining the results of pvaf and wave methods, in order to benefit from their strengths and overcome their complementary weaknesses. In principle, their integration allows to remove from low to high frequency PA contributions and at the same time eliminates the risk of removing physiological components.

Regarding the two signal lengths for ICA calculation, i.e. the whole dataset or only the PA intervals, both of them led to acceptable results and none outperformed the other, as determined by the quantitative comparisons. The variability in the performance of the selection methods across the subjects of the group was hypothesized to be related to the overall quality of the EEG acquisition that in turn influences the goodness of the detection of the R peaks and the performance of OBS and ICA decomposition.

### Methodological limitations

The setting of proper parameters represents a crucial step that influences the performance of each selection method. The parameters chosen in our study led to the removal of almost one third of the components in the corr, wave and pacf methods, a higher percentage with respect to the pvaf method. Nevertheless, the latter influenced the original signal more than the others, indicating that the information about which components are removed is more relevant than the number itself.

In the variance contribution method, the 2.5% threshold was chosen based on empirical observations: when using higher thresholds, such as 5%, only a few components were removed (the first sorted by energy), which typically did not fully resemble the artefact. Instead, the 2.5% threshold setting also allowed to remove cardiac related artefacts with lower energy. It is worth mentioning that the interval used for computing the explained variance has an effect on the results and in turn influences the choice of an appropriate threshold. In our study, the fact that the highest variance contributions were found in the first components indicates that our PA interval (from 0 to 700 ms after the R peaks) did not always match the PA occurrence. On the other hand, the high temporal and spatial variability of the PA within and between subjects makes the choice of an interval appropriate for all subjects very challenging.

In the selection based on correlation, two factors are determinant, i.e. the quality of the ECG signal or template and the correlation threshold. In our study, we decided to use an artefact template instead of the ECG signal, since the latter seemed to be different from the artefact occurrences in the EEG signal; nevertheless, from our results we could deduce that it is possible that our template did not resemble the cardiac artefact, at least in the majority of subjects. The reason for this could be that the template was estimated from the EEG signal before OBS correction. Regarding the thresholds, the choices between absolute or relative thresholds and of the threshold value are not trivial.

The selection method based on wavelets has the major drawback of being manual, relying on the user’s ability to recognize the PA frequency contributions. To optimize the quality of PA removal, the user has to train himself to inspect the IC signals together with their time-varying spectral content. In the current application, the performances of the wave method were not optimal, because the low frequency contribution of the PA was not removed as efficiently as when using the pvaf method. After proper training, the user may be able to identify all the artefactual components. Either way, the combination of visual inspection and quantitative indices for the selection of components would be very beneficial.

In the selection method based on the ICs PACF, the most delicate step was related to the setting of the threshold, at least in our application. Since many ICs had a peak in correspondence of the R–R distance, we decided to remove only the ones with a peak amplitude above a certain threshold, i.e. one third of the maximum amplitude. A comparison between different thresholds would also be useful in this case.

It is worth mentioning that the overall performance of the selection methods strongly depends on the quality of the ICA decomposition. In this study we used the Infomax ICA algorithm, which was proved to be effective when used for PA correction in [31], with the extended option, allowing for components with negative kurtosis. Nevertheless, in the majority of datasets the ICs signals changed characteristics over time, sometimes mixing timeframes of cardiac-related activity with others of neuronal activity. Debener et al., [35] suggested that the distortion of ICA solutions might increase with the MR scanner static magnetic field. Although the reason of the failure of ICA estimation is still unknown, it could be partially ascribed to the unmet assumption of spatial stationarity of the sources. Besides the importance of a proper selection criterion, the quality of ICA decomposition is of primary importance for obtaining satisfactory results.

## Conclusion

A full exploitation of the potentials of EEG-fMRI integration is possible only if an optimal cleaning of the EEG signal from the MR related artefacts is performed. The cardiac-related artefact has variable characteristics over space and time that make it difficult to remove. This study focused on the PA correction based on the combination of OBS and ICA and compared eight different ICA corrections, i.e. two intervals for the ICA calculation and four methods for selecting the PA-related components. Different criteria for the assessment of the quality of PA removal were used, some sensitive to the artefact removal, others also to the preservation of the information of interest. The two intervals of ICA calculation led to similar results, whereas the selection of the artefactual components based on their wavelets transform emerged as preferable to the other selection methods, since it resulted in the ability to highlight the PA-related components, making them easily distinguishable from the neuronal ones. The results were usually in agreement across the two datasets, thus confirming the reproducibility of the performance of each ICA correction algorithm. Even though the quality of the PA removal largely depends on the performance of the ICA decomposition, the present work provides valuable information on the optimization of the selection of PA-related ICs and on the assessment of the effects that each PA correction has on the EEG signal.
